# Post-translational modifications of p65: state of the art

**DOI:** 10.3389/fcell.2024.1417502

**Published:** 2024-07-10

**Authors:** Xutao Sun, Shuo Cao, Caiyun Mao, Fengqi Sun, Xuanming Zhang, Yunjia Song

**Affiliations:** ^1^ Department of Typhoid, School of Basic Medical Sciences, Heilongjiang University of Chinese Medicine, Harbin, China; ^2^ Department of Pharmacology, School of Basic Medical Sciences, Heilongjiang University of Chinese Medicine, Harbin, China; ^3^ Department of Pathology, School of Basic Medical Sciences, Heilongjiang University of Chinese Medicine, Harbin, China; ^4^ Department of Orthopedics, The Second Affiliated Hospital of Harbin Medical University, Harbin, China

**Keywords:** NF-κB p65, post-translational modifications, inflammation, immune response, transactivation activity

## Abstract

P65, a protein subunit of NF-κB, is a widely distributed transcription factor in eukaryotic cells and exerts diverse regulatory functions. Post-translational modifications such as phosphorylation, acetylation, methylation and ubiquitination modulate p65 transcriptional activity and function, impacting various physiological and pathological processes including inflammation, immune response, cell death, proliferation, differentiation and tumorigenesis. The intricate interplay between these modifications can be antagonistic or synergistic. Understanding p65 post-translational modifications not only elucidates NF-κB pathway regulation but also facilitates the identification of therapeutic targets and diagnostic markers for associated clinical conditions.

## 1 Introduction

**FIGURE 1:**
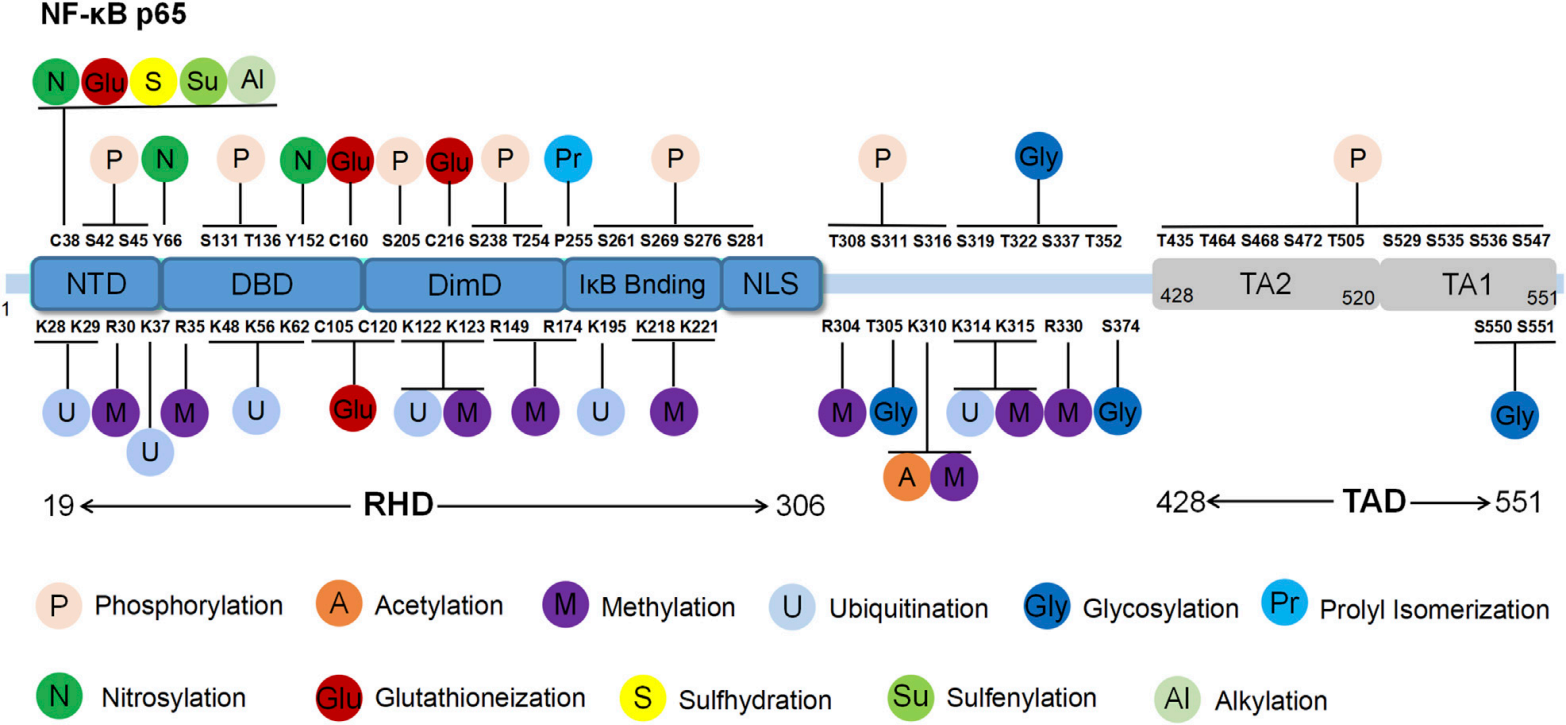
Structure and post-translational modification sites of the human p65 protein. NTD: N-terminal domain; DBD: DNA binding domain; DimD: dimerisation domain; NLS: nuclear localisation sequence.

NF-κB, a pivotal transcription factor ubiquitous across in nearly all ccell types, orchestrates diverse biological processes including inflammation, immunity, differentiation, cell growth, tumour development and apoptosis. The NF-κB protein family predominately reside in the cytoplasm across diverse cell types. By modulating gene expression, the members play a regulatory role in various physiological and pathological processes such as programmed cell death, inflammation, immune responses, cell proliferation, differentiation and tumorigenesis (Gaptulbarova et al., 2020). The mammalian NF-κB family comprises five members: p65 (NF-κB3/RelA), p105/p50 (NF-κB1), P100/P52 (NF-κB2), RelB and c-Rel. Among these, the heterodimer formed by p65 and p50, facilitated by their respective Rel homology domains (RHD) near the amino terminus, is extensively studied (May and Ghosh, 1997; Giridharan and Srinivasan, 2018). P65, a 65 kDa peptide with 551 amino acids, is widely distributed in eukaryotic cells. The RHD domain of the p65 protein, along with its acidic transactivation domain (TAD) at the carboxyl terminus, is involved in the regulation of p65 target genes (Figure 1). Notably, dysregulation of its mechanisms can lead to the occurrence of various diseases, including cancer (Leeman, et al., 2008).

The RHD domain is located between the 19th and 306th amino acids of p65, exhibiting a beta-barrel secondary structure that is characteristic of the Rel family. Through crystallography techniques, the RHD domain was reported to encompass subdomains including the N-terminal domain (NTD), DNA binding domain (DBD), dimerisation domain (DimD), an inhibitory molecule IκB binding site and a nuclear localisation sequence (NLS) subdomain (Malek et al., 2003). The NLS subdomain is responsible for DNA binding, dimerisation and nuclear translocation. In the resting state, IκB binds to the NLS domain, obstructing p65’s interaction with target genes and preventing it from recognising activation signals, thereby maintaining an inactive state. External stimuli trigger IκB degradation, exposing the NLS region and enabling p65 nuclear translocation to regulate gene expression.

The TAD domain, located between the 428th and 551st amino acids, primarily regulates the transcriptional activity of p65. TAD is a bipolar domain rich in acidic amino acids and comprises two subdomains, namely, TA1 and TA2. TA1 is positioned between the 520th and 549th amino acids near the C-terminus, while TA2 spans from the 428th to 520th amino acids, existing between NLS and TA1. The interactions and functional coordination of these structural domains enable NF-κB p65 to regulate the various signalling networks and biological processes such as gene transcription, cell proliferation, apoptosis and inflammation. Understanding the structural and functional intricacies of NF-κB p65 sheds light on the regulatory mechanisms underlying NF-κB pathway activation, offering insights into potential therapeutic strategies for related diseases (Ngo et al., 2020).

As an integral component of the NF-κB signaling pathway, p65 assumes a critical function in the modulation of immune responses and inflammatory processes. Specifically, p65 governs the transcriptional regulation of various cytokines and cell surface receptors within the immune system, thereby facilitating the activation of T cells. This activation is imperative for the maturation of T cells and the regulation of their survival and functionality (Kane et al., 2002). Moreover, p65 is essential for the proper functioning of B cells, as aberrant regulation of this subunit may precipitate B cell-related pathologies (Brownlie et al., 2023). Additionally, p65 is indispensable for the innate immune response, as it influences the activities of macrophages and dendritic cells (Granelli-Piperno et al., 1995; Dorrington and Fraser, 2019). These functions play a crucial role in facilitating cellular defenses against bacterial, viral, and other pathogenic agents. P65 has been identified as a key regulator of cytokines and chemotactic factors, facilitating the activation of various cellular factors including IL-1β, TNF-α, and chemotactic factors, which in turn promote the migration of immune cells to sites of infection and inflammation, thereby bolstering the immune response (Garcia et al., 2000). In the context of inflammation, p65 governs the expression of a range of pro-inflammatory cytokines, such as TNF-α and various interleukins (e.g., IL-1β, IL-6), which stimulate local tissue inflammatory reactions and elicit antigen-specific immune responses (Zhang et al., 2005; Wang et al., 2015). P65 is shown to upregulate the expression of adhesion molecules, including ICAM-1 and VCAM-1, on endothelial cells, facilitating the adhesion and transmigration of inflammatory cells across the vascular endothelium into inflamed tissues (Shu et al., 1993; Jahnke and Johnson, 1994). Chronic inflammatory responses are complex phenomena characterized by the interplay of multiple inflammatory mediators and cellular interactions. P65 plays a crucial role in sustaining the chronic inflammatory milieu by modulating these diverse components of the inflammatory response or the associated hypoxic conditions (Fitzpatrick et al., 2011; Morein et al., 2021). In autoimmune diseases and chronic inflammation, the overactivation of the p65 transcription factor is frequently linked to the perpetuation and worsening of symptoms. This includes prolonged activation of inflammatory cells, heightened cytokine production, tissue injury, and fibrosis. Additionally, p65 plays a role in cell survival and apoptosis by stimulating anti-apoptotic genes like Bcl-2, as well as regulating the cell cycle and impacting cell proliferation (Bae et al., 2003; Moles et al., 2016).

Moreover, post-translational modifications, subcellular compartmentalisation mechanisms and interactions with other co-factors or co-repressors also regulate the activity of p65. Dysregulation of p65 is implicated in various pathological conditions such as chronic inflammation, neurodegenerative disorders, tumours, atherosclerosis and immune deficiencies (Lawrence, 2009). Phosphorylation, acetylation, methylation, ubiquitination and other modifications at p65 intricately regulate its transcriptional activity, influencing its subcellular localisation, DNA binding and overall function (Perkins, 2006). Therefore, an in-depth exploration of the post-translational modifications of the p65 protein holds promise for elucidating immune and inflammatory responses and identifying potential therapeutic targets for associated diseases. This review presents the structure and function of p65, along with its post-translational modification mechanisms and regulatory processes. Furthermore, it summarises the relationship between modification sites and modification types.

## 2 Phosphorylation of p65

**FIGURE 2:**
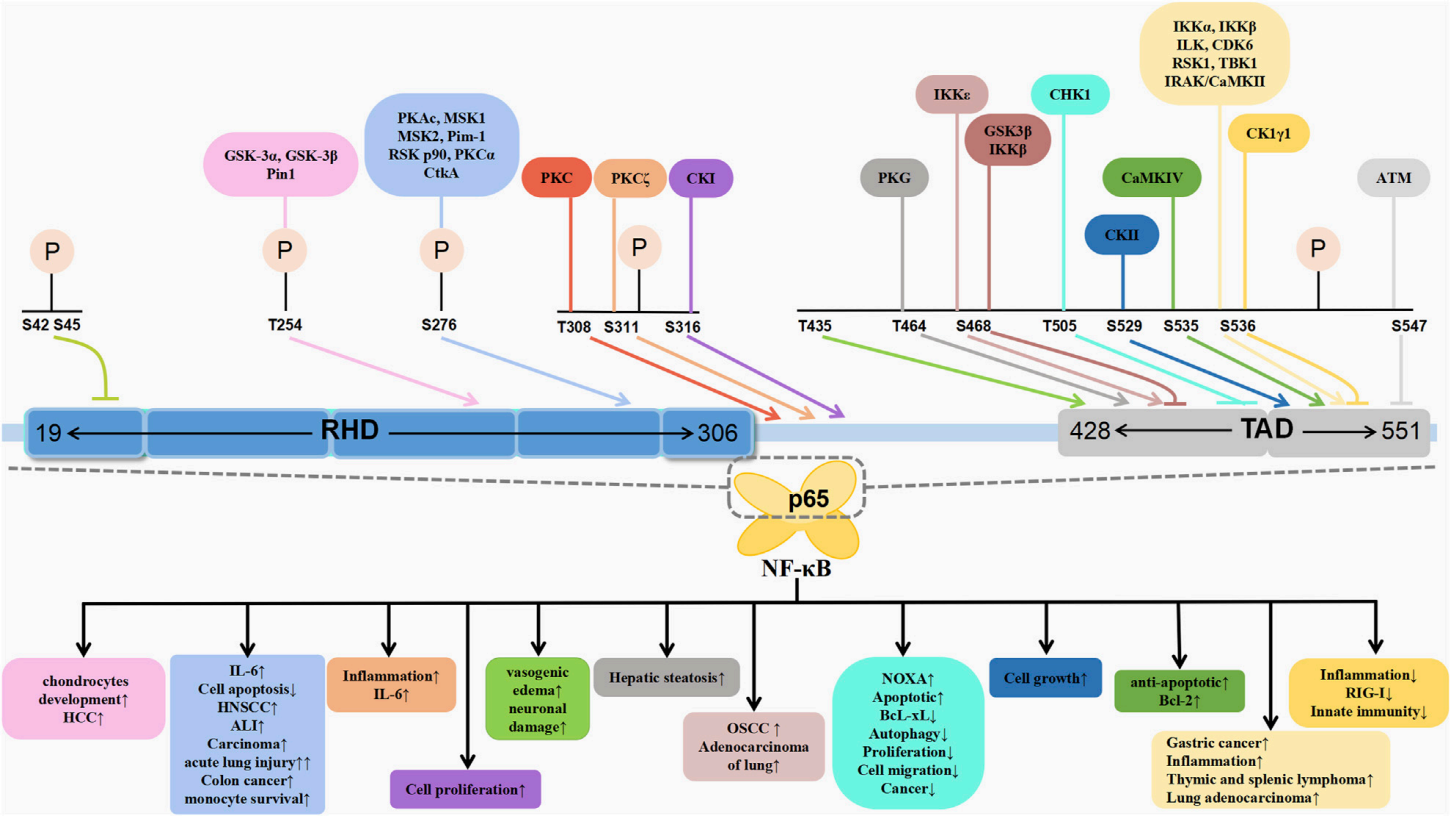
Schematic association of phosphorylation of p65 with associated diseases.

**FIGURE 3:**
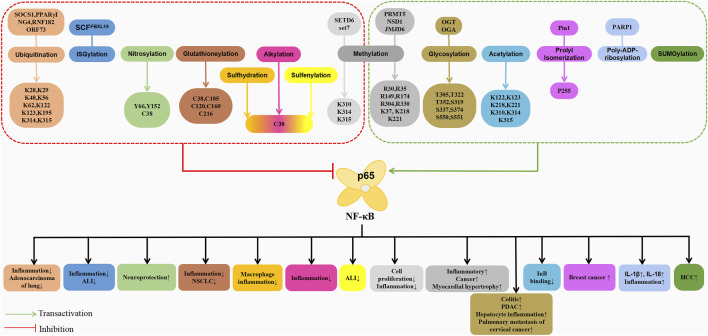
Schematic association of post-translational modifications (in addition to phosphorylation) of p65 with associated diseases.

The post-translational modifications of p65 intricately regulate NF-κB’s transcriptional activation, profoundly influencing the onset and progression of associated diseases (Table 1). Current research predominantly emphasises the phosphorylation modifications of p65, encompassing the entire signalling transduction pathway (Figure 2). Additionally, acetylation, ubiquitination and other forms of modifications have garnered attention. The dysregulation of p65 post-translational modifications can disrupt NF-κB signalling, contributing to the development of various malignancies and rendering it a significant therapeutic target (Figure 3). Current drugs targeting NF-κB p65 modifications include Kegan Liyan oral liquid, geniposide and baicalin (Feng et al., 2014; Shi et al., 2014; Zhang et al., 2016). Phosphorylation of p65 has emerged as a focal point in NF-κB post-translational modification research. Currently, 18 serine and 5 threonine phosphorylation sites have been identified on p65, alongside several associated kinases. Phosphorylation at these sites modulates the transcriptional levels of p65, either activating or inhibiting the NF-κB pathway. Moreover, various stimuli induce p65 phosphorylation through distinct pathways, prompting cytoplasmic and nuclear transformations and altering subcellular localisation and biological functions. Overall, the phosphorylation of p65 significantly regulates NF-κB activity.

**TABLE 1:** Post-translational modifications of p65.

Modifications	Sites	Enzyme	NF-κB pathway	Effects	References
Phosphorylation	S276	PKAc	Transactivation	—	Jamaluddin et al. (2007)
	S276	MSK1	Transactivation	IL-6↑	Vermeulen et al. (2003)
	S276	MSK2	Transactivation	—	Vermeulen et al. (2003)
	S276	Pim-1	Transactivation	IL-6, TNF-α↑	Nihira et al. (2010)
	S276	Pim-1	Transactivation	Acute lung injury↑	Wang J. et al. (2019)
	S276	CtkA	Transactivation	—	Kim et al. (2010)
	S276	RSK p90	Transactivation	Monocyte survival↑	Wang et al. (2010)
	S276	PKCα	Transactivation	Monocyte survival↑	Wang et al. (2011)
	T254	Pin1	Transactivation	—	Shinoda et al. (2015)
	T254	GSK-3α, GSK-3β	Transactivation	Chondrocytes development↑	Itoh et al. (2012)
	S205, S281	—	Transactivation	—	Anrather et al. (2005)
	S131, T136, S238, S261, S269, S472	—	—	—	Lanucara et al. (2016)
	S42, S45	—	Inhibition	—	Lanucara et al. (2016)
	S536	IKKα, IKKβ	Transactivation	—	Sakurai et al. (1999)
	S536	TBK1, IKKε	Transactivation	—	Mattioli et al. (2006)
	S536	ILK	Transactivation	—	Ahmed et al. (2014)
	S536	RSK1	Transactivation	—	Bohuslav et al. (2004)
	S536	CDK6	Transactivation	Thymic/splenic lymphoma proliferation↑	Buss et al. (2012)
	S536	GSK-3β	Transactivation	—	Cheng et al. (2021)
	S536	IRAK/CaMKⅡ	Transactivation	—	Kim et al. (2014)
	S536	CK1γ1	Inhibition	Inflammation↓, RIG-I↓ Innate immunity↓	Wang et al. (2014)
	S535	CaMKIV	Transactivation	Anti-apoptotic↑, Bcl-2↑	Bae et al. (2003)
	S547	ATM	Inhibition	—	Sabatel et al. (2012)
	S529	CKII	Transactivation	Cell growth↑	Wang et al. (2000)
	T505	CHK1	Inhibition	BcL-xL↓, NOXA↑, cell apoptotic↑, cell autophagy↓ cell proliferation↓ cell migration↓	Msaki et al. (2011)
	T505	CHK1	Inhibition	Apoptosis↑, liver cells proliferation↓	Moles et al. (2016)
	S468	GSK3β	Inhibition	Proliferation and tumorigenesis of lung adenocarcinoma↑	Buss et al. (2004)
	S468	GSK3β	Transactivation	—	Cheng et al. (2021)
	S468	IKKε	Transactivation	—	Mattioli et al. (2006)
	S468	IKKβ	Inhibition	—	Schwabe and Sakurai. (2005)
	S468	—	—	OSCC proliferation↑	Zhang et al. (2021)
	T435	—	Transactivation	Vasogenic edema↑ neuronal damage↑	Kim et al. (2012)
	T464	PKG	Transactivation	Hepatic steatosis↑	Zhou et al. (2013)
	S311	PKCζ	Transactivation	IL-6↑, inflammation↑	Yao et al. (2010)
	S316	CKI	Transactivation	Cells proliferation↑	Wang et al. (2015)
	T308	PKC	—	—	Ma et al. (2017)
Acetylation	K218, K221, K310	—	Transactivation	IκB binding↓	Chen et al. (2002)
	K122, K123	—	Transactivation	IκB binding↓	Kiernan et al. (2003)
	K314,K315	—	—	—	Buerki et al. (2008)
Methylation	R30, R35, R174, R304, R330	PRMT5	Transactivation	—	Harris et al. (2014)
	K310	SETD6	Inhibition	Cells proliferation↓, inflammatory↓	Levy et al. (2011)
	K314, K315	set7	—	—	Yang et al. (2009)
	K37	—	Transactivation	Inflammatory↑	Ea and Baltimore. (2009)
	K218, K221	NSD1	Transactivation	Cancer cell proliferation↑	Lu et al. (2010)
	R149	JMJD6	Transactivation	Myocardial hypertrophy↑	Guo et al. (2023)
Ubiquitination	—	SOCS1	Inhibition	—	Maine et al. (2007)
	K28	PPARγ	Inhibition	Xenograft tumours↓	Hou et al. (2012)
	K48, K62	ING4	Inhibition	Inflammation↓	Hou et al. (2014)
	—	RNF182	Inhibition	PDL1 transcription and immune suppression in lung adenocarcinoma↓	Zeng et al. (2023)
	—	ORF73	Inhibition	—	Rodrigues et al. (2009)
	K122	FBXW2	Inhibition	SOX2↓ breast cancer cell proliferation and migration↓	Ren et al. (2022)
	K122, K123, K314, K315, K56	—	Inhibition	—	Li et al. (2012)
	K29	TRAF7	Inhibition	Cells death↑	Zotti et al. (2011)
	K195	—	Inhibition		Fan et al. (2009)
Glycosylation	T322, T352	—	Transactivation	IκB binding↓	Yang et al. (2008)
	T322, T352	—	Transactivation	Colitis susceptibility↑	Yang et al. (2015)
	T305, S319, S337, T352, S374	—	—	—	Ma et al. (2017)
	S550, S551	OGT	Transactivation	PDAC proliferation↑	Motolani et al. (2023)
	—	—	Transactivation	oxidative stress and inflammation in hepatic cells↑	Lee et al. (2019)
	—	—	Transactivation	cervical cancer cells metastasis↑	Ali et al. (2017)
Poly-ADP-ribosylation	—	PARP1	Transactivation	IL-1β↑, IL-18↑, inflammation↑	Liu et al. (2012)
S-nitrosylation	Y66, Y152	—	Inhibition	—	Park et al. (2005)
	C38	—	Inhibition	—	Kelleher et al. (2007)
	—	—	Inhibition	Neuroprotective ↑	Caviedes et al. (2021)
Glutathioneylation	C38, C160, C216	—	Inhibition	—	Li and Verma. (2002)
	—	—	Inhibition	Pulmonary inflammation↓, hypoxic↑, apoptosis↑	Qanungo et al. (2007)
	—	—	Inhibition	OS in NSCLC↓	Zhang et al. (2023)
S-sulfhydration	C38	—	Inhibition	anti-apoptotic↑	Sen et al. (2012)
	C38	—	Inhibition	Macrophage inflammation↓	Du et al. (2014)
S-sulfenylation	C38	—	Inhibition	Acute lung injury↓	Chen et al. (2017)
Prolyl Isomerization	P255	Pin1	Transactivation	—	Ryo et al. (2003)
ISGylation	—	SCF^FBXL19^	Inhibition	Lung inflammation↓ Lcute lung injury↓	Li et al. (2023)
SUMOylation	—	—	Transactivation	HCC invasion and metastasis↑	Liu et al. (2020)
Alkylation	C38	—	Inhibition	Inflammation↓	García-Piñeres et al. (2004)

OSCC: oral squamous cell carcinoma; PDAC: pancreatic ductal adenocarcinoma; OS: overall survival; NSCLC: non-small cell lung cancer; HCC: hepatocellular carcinoma.

### 2.1 Phosphorylation sites within RHD

Within the RHD domain, S276 is the first identified phosphorylation site on p65, mediated by various kinases. The principal kinase implicated in p65 phosphorylation is the catalytic subunit PKAc of protein kinase A (PKA). Zhong et al. (1997) demonstrated that PKAc maintains an inactive state by binding to IκB-α and IκB-β within the NF-κB-IκB complex through non-covalent interactions. Upon induction by NF-κB activators such as LPS, IκB degradation activates PKAc bound to it in a cAMP-independent manner, followed by p65 phosphorylation at Ser276. Notably, PKAc-mediated phosphorylation from the NF-κB-IκB complex effectively enhances NF-κB transcriptional activity without affecting its nuclear translocation and DNA binding. Tumour necrosis factor-α (TNF-α) stimulation of monocytes also promotes PKAc-mediated phosphorylating of the S276 residue of p65, which is a process dependent on oxidative stress, resulting in reduced NF-κB transcriptional activity in the absence of ROS (Jamaluddin et al., 2007). In addition to PKAc, mitogen- and stress-activated protein kinase-1 (MSK1) phosphorylates p65 in a ROS-dependent manner, highlighting the critical role of pro-inflammatory cytokines/chemokines expression (Xue et al., 2023). In mouse fibroblasts treated with TNF-α, MSK1 phosphorylated the S276 residue of NF-κB p65 in the nucleus, enhancing the transcriptional activity of NF-κB and IL-6, although ROS clearance can hinder the interaction between MSK1 and p65 (Vermeulen et al., 2003; Xue et al., 2023). Furthermore, in human MDA-MB-231 cells, UV-induced transactivation of p65 primarily relies on MSK2 rather than MSK1 for phosphorylation at the p65 S276 site, positively regulating NF-κB activity in promoting cell survival (Vermeulen et al., 2003). PKAc phosphorylates S276 in the cytoplasm, while MSK1/2-mediated phosphorylation occurs in the nucleus. Studies have revealed that the PKA inhibitor H-89 (Arun et al., 2009) and curcumin analogue UBS109 (Zhu et al., 2014) can inhibit the phosphorylation of p65 at S276 by downregulating PKAc, thereby inhibiting the growth of squamous cell carcinoma (SCC) in the head and neck region and highlighting its therapeutic potential in head and neck SCC.

The protein kinase Pim-1, known for phosphorylating serine and threonine residues, plays a pivotal role in tumorigenesis. Upon TNF-α stimulation, Pim-1 rapidly phosphorylates p65 at S276, counteracting the suppressor of cytokine signalling 1 (SOCS1)-dependent ubiquitination and subsequent degradation. This Pim-1-induced phosphorylation of p65 at S276 contributes to the stabilisation and activation of p65, consequently protecting cells from TNF-α-induced apoptosis (Nihira et al., 2010). Wang J. et al. (2019) reported that pretreatment with the Pim-1 inhibitor SMI-4a reduced the nuclear upregulation of p65 and the LPS-induced phosphorylation of S276, suggesting the S276 kinase pathway as a potential therapeutic target for LPS-induced acute lung injury. Additionally, the pro-inflammatory protein JHP940 from *Helicobacter pylori* strain J99, also known as cell translocating kinase A (CtkA), exhibits serine/threonine kinase activity, translocating into cells and upregulating NF-κB activity in a kinase-dependent manner. Further experiments revealed that recombinant CtkA significantly enhances the phosphorylation of p65 at S276 in human epithelial cell carcinoma, thereby indirectly affecting NF-κB activity through upstream components (Kim et al., 2010). Thus, understanding this regulatory mechanism offers insights into the inflammatory processes during *H. pylori* infection. Phosphorylation of the highly conserved S276 residue induces conformational changes in p65, facilitating enhanced recruitment of the transcriptional coactivators p300/CBP, thereby enhancing the transcriptional activity of p65 (Nihira et al., 2010; Feng et al., 2014). Anti-inflammatory substances such as Licochalcone A and IH-1 (5-(1H-indole-3-ylmethylene) imidazolidine-2,4-dione) inhibit S276 phosphorylation, preventing interaction between the p65 subunit and p300/CBP, thereby reducing NF-κB transactivation and exerting anti-inflammatory effects (Furusawa et al., 2009; Lin et al., 2021).

Furthermore, other kinases that catalyse the phosphorylation of p65 at S276, such as ribosomal S6 kinase (RSK) p90 and protein kinase C (PKC), also exist. RSK p90, a downstream target kinase of ERK1/2, mediates the phosphorylation of p65 induced by protease-activated receptors (PARs). PAR1 and PAR2 activation induces RSK1 and RSK2’s nuclear translocation, wherein RSK1 inhibition reduces PAR2-induced p65 phosphorylation. Similarly, RSK2 inhibition blocks PAR1-induced p65 phosphorylation. However, post-PAR activation reduced the binding between histone deacetylase 2 (HDAC2) and p65, thereby increasing NF-κB transcriptional activity (Wang et al., 2010). This mechanism holds clinical significance for inflammatory bowel disease and colon cancer. Notably, PKCα is an upstream activator of NF-κB. In human and murine macrophages, the macrophage colony-stimulating factor induces the activation of PKCα, which, in turn, leads to its phosphorylation of p65 at S276. This enhances the transcriptional activity of NF-κB and regulates the survival of monocytes (Wang et al., 2011).

Phosphorylation of the threonine residue T254 within the RHD domain, in addition to serine, can be induced by TNF-α stimulation. This phosphorylation (Thr254-Pro motif) recruits the peptidyl-prolyl cis-trans isomerase NIMA-interacting 1 (Pin1), which inhibits p65-IκBα interaction, stabilising p65 and enhancing its nuclear accumulation and transcriptional activation, which, in turn, is achieved by blocking SOCS-1-mediated ubiquitination of p65 and its ubiquitin-mediated proteolysis (Ryo et al., 2003).

Following the binding of the phosphorylated Thr254-Pro motif within p65, Pin1 induces S276 phosphorylation in p65, thereby regulating p56 DNA binding. Knocking out Pin1 was found to significantly inhibit the expression of phosphorylated T254 and S276 in p65, leading to a reduction in NF-κB activity in HepG2 cells, which, in turn, inhibits the progression of hepatocellular carcinoma (HCC) (Shinoda et al., 2015). Additionally, Glycogen synthase kinase-3 (GSK-3), comprising GSK-3α and GSK-3β, phosphorylates p65 at T254, a process crucial for the early chondrocyte development. Mice lacking GSK-3α and GSK-3β exhibit impaired chondrocyte differentiation and dwarfism, owing to impaired phosphorylation at p65 (Itoh et al., 2012).

Anrather et al. (2005) identified multiple phosphorylation sites on p65, with varying degrees of phosphorylation at various serine residues. Among these, S205 was identified as the site with the highest phosphorylation level, suggesting that serine phosphorylation at 205 is necessary for other phosphorylation reactions to occur. Furthermore, compensatory phosphorylation at other sites indicates that the loss of phosphorylated serine at a single site does not affect the overall DNA binding activity of p65. Luciferase reporter assays also demonstrated that mutation at a single site is insufficient to completely inactivate p65, with mutations at specific sites only impacting certain stimuli. This implies that differential phosphorylation at multiple sites on p65 could be the reason it targets specific gene subsets for a specific response to different cellular signals.

Lanucara et al. (2016) employed a combination of shotgun and targeted mass spectrometry strategies to delineate the phosphorylation time kinetics of endogenous p65 in SK-N-AS neuroblastoma cells in response to TNF-α stimulation. The study identified multiple phosphorylation sites on p65, including seven novel sites (Ser42, Ser131, Thr136, Ser238, Ser261, Ser269 and Ser472). Specifically, phosphorylation levels of Ser45 and Ser238 induced by PKA, as well as Thr136, Ser131 and Ser311 induced by IKKβ, significantly increased in the presence of p50 (i.e., the heterodimeric partner of p65) but significantly decreased upon the addition of IκBα. This suggests that in a steady-state environment, several phosphorylation sites on p65 within the NF-κB-IκB complex are shielded by IκB or exhibit reduced accessibility after binding to IκB due to conformational changes, indicating the differential regulation of distinct p65 dimers for functional specificity. Furthermore, Ser45 functions as the primary cellular phosphorylation site, and the phosphorylation of Ser42 and/or Ser45 negatively impacts p65 transcriptional activity by reducing its DNA binding.

### 2.2 Phosphorylation sites within TAD

The S536 residue in the TA1 subdomain of p65 has garnered substantial attention for its phosphorylation by various kinases, influencing downstream biological functions. The phosphorylation at p65’s S536 position was first reported by Sakurai et al. (1999), utilising *in vitro* kinase assays. Utilising immunoprecipitation methods, Sakurai et al. identified IKKα and IKKβ as key kinases responsible for phosphorylating p65 at S536, altering its conformation and enhancing transcription. TANK binding kinase (TBK1), a homolog of IKKε, requires the phosphorylation of a key serine residue within its activation loops to maintain kinase activity. TBK1 has also been identified as a kinase for p65 phosphorylation at S536 (Mattioli et al., 2006). Thus, IKKε and TBK1 significantly influence the constitutive p65 phosphorylation levels at S536 (Adli and Baldwin, 2006).

Integrin-linked kinase (ILK) is a widely expressed and highly conserved serine/threonine protein kinase associated with cancer progression. While ILK does not participate in the LPS-induced classical NF-κB signalling pathway, it mediates p65 S536 phosphorylation in response to LPS as an alternative activation pathway for NF-κB signalling. A similar scenario occurs in gastric cancer cells infected with *H. pylori*, wherein ILK regulates the phosphorylation induced by *H. pylori*, promoting TNF-α secretion via the PI3K/Akt pathway (Ahmed et al., 2014). Upon treatment with the anticancer drugs doxorubicin and etoposide, Saos-2-Tet-On-p53 cells exhibited an upregulation of the tumour suppressor p53, which is also involved in the MEK1/RSK1 pathway. This activation leads to the translocation of activated RSK1 kinase from the cytoplasm to the nucleus, where it phosphorylates the p65 subunit at S536. Consequently, this phosphorylation results in the reduction of the nucleus-to-cytoplasm shuttling of the NF-κB/IκBα complex, enhancement of NF-κB binding to homologous kappaB enhancers and increase in the transcriptional expression of downstream NF-κB-related genes (Bohuslav et al., 2004). Similarly, angiotensin II (AngII) can increase the activity of RSK kinase, enhancing the phosphorylation of the p65 S536 site in vascular smooth muscle cells through the Ras/MEK1/ERK/RSK signalling pathway, a process that is dependent on both time and AngII concentration. The activation of NF-κB by AngII and the subsequent production of pro-inflammatory factors such as IL-6 have been identified as the underlying causes of vascular inflammation (Zhang et al., 2005). Cyclin-dependent kinase6 (CDK6), a member of the cyclin-dependent kinase 2 (CDK2)-related kinase family, is often overexpressed in human tumours. Buss et al. (2012) reported that CDK6 directly regulates p65 phosphorylation in the cytoplasm and nucleus, identifying it as an S536-specific NF-κB kinase. In an Eμ-v-cyclin transgenic mouse model, increased levels of CDK6, phosphorylated p65 S536, NF-κB activity and thymic and splenic lymphoma formation suggest that CDK6 promotes tumorigenesis through NF-κB. Furthermore, in lung adenocarcinoma, GSK-3β was found to phosphorylate S536 and activate NF-κB transcription (Cheng et al., 2021).

EB virus latent membrane protein 1 (LMP1) induces p65 S536 phosphorylation through interleukin-1 (IL-1) receptor-associated kinase 1 (IRAK1). Notably, the kinase activity of IRAK1 is not essential for LMP1-induced NF-κB activation, thereby highlighting IRAK1’s role as a scaffold protein in the recruitment of p65 S536 kinase to enhance NF-κB-dependent transcriptional activity. In the LMP1-induced NF-κB activation pathway, IRAK1 interacts with CaMKII and mediates CaMKII activation, which, in turn, phosphorylates the S536 site in the cytoplasm to activate NF-κB, thereby participating in the process of EBV-induced transformation of proliferating lymphoblastoid cells (LCL) (Kim et al., 2014; Song et al., 2006). While several studies indicate that the phosphorylation of p65 S536 plays an activating role in transcription, there exists a small fraction of studies claiming that phosphorylated p65 S536 acts as a negative regulator of the NF-κB pathway, preventing inflammation by inhibiting NF-κB signal transduction (Wang et al., 2014; Pradère et al., 2016). Specifically, members of the casein kinase 1 (CK1) family, such as CK1γ1, inhibit NF-κB transcriptional activity and downstream pro-inflammatory factors production by phosphorylating p65 S536, thereby suppressing the RIG-I signalling pathway activated by RNA viruses like the Sendai virus and its mediated antiviral response, serving as a negative regulator of innate immunity (Wang et al., 2014).

Phosphorylation of p65 at position 65 is a reversible process, with phosphates playing a crucial role in restoring NF-κB responsiveness and inactivating NF-κB signalling upon the removal of stimuli or target gene expression. Consequently, the dephosphorylation of p65 plays a significant role in the regulation of NF-κB transcriptional activation. Chew et al. (2009) demonstrated through a genome-scale siRNA screening that the dephosphorylating enzyme WIP1 can dephosphorylate S536, thereby reducing the interaction between p65 and p300 and exerting a negative regulatory effect on NF-κB. Similarly, the protein phosphatase 2A (PP2A) was identified as a dephosphorylating enzyme for p65, revealing specificity in dephosphorylating S536 and inhibiting NF-κB transcriptional activity (Yang et al., 2001; Li et al., 2006). Additionally, a halogenase-dehalogenase (HAD) family serine phosphatase, SerB, secreted by Porphyromonas gingivalis, specifically dephosphorylated S536 of p65 in gingival epithelial cells. SerB binds to p65 and colocalises with it in the cytoplasm, promoting its dephosphorylation and inhibiting TNF-α-induced nuclear translocation of p65 in epithelial cells, thereby regulating host inflammatory pathways and inhibiting innate immunity at mucosal surfaces (Takeuchi et al., 2013).

Research has indicated that the residue S535, adjacent to S536, can be phosphorylated by Calcium/Calmodulin-dependent protein kinase IV (CaMKIV). Co-expression of active CaMKIV with wild-type p65 has shown the restoration of etoposide-induced cell apoptosis and an increase in Bcl-2 protein levels, suggesting that the phosphorylation of NF-κB p65 by CaMKIV on S535 enhances the expression of NF-κB target genes, including anti-apoptotic genes, thereby inhibiting cell apoptosis (Bae et al., 2003). Etoposide-like drugs induce DNA double-strand breaks (DSBs), triggering the activation of the DNA damage response signalling pathway. Within this pathway, Ataxia telangiectasia mutated (ATM), a nuclear kinase that mediates DNA damage response upon DSB formation, binds to the N-terminus of p65 and specifically phosphorylates the S547 site, facilitating its interaction with HDAC1 without affecting DNA binding and ultimately downregulating the transcription of NF-κB downstream genes (Sabatel et al., 2012). Additionally, the residue S529 within the TA1 region, adjacent to the TA2 domain, is phosphorylated by CKII, a modification inhibited by the binding of IκBα to p65. Upon degradation of IκBα, CKII enhances NF-κB transcriptional activity through the modification of p65 at S529, exerting regulatory control over cell growth (Wang et al., 2000). Additionally, the dysregulation of the NF-κB pathway with ageing has been reported to exhibit higher NF-κB levels in the monocyte nuclei of older individuals compared to younger ones, a difference that can be attributed to the phosphorylation of p65 at S529 (Tavenier et al., 2020).

Within the TA2 subregion, phosphorylation can also occur at the T505 site. ARF, a tumour suppressor, is a core component of cellular defence against the activation of oncogenes. CHK1 is a kinase that exists downstream of the ATM- and Rad3-related (ATR) kinase. Phosphorylation induced by CHK1 can occur through ARF mediation, wherein the T505-modified p65 is predominantly located in the nucleus (Rocha et al., 2005). Other inducers have been unable to phosphorylate T505, indicating that the adaptor protein induced by ARF is vital for CHK1 binding to specific substrates. The activation of ATR kinase and CHK1 by ARF differs mechanistically and functionally. Following treatment with the anticancer drug cisplatin, DNA damage occurs in cells, and the phosphorylation of T505 results in the transcriptional repression of the NF-κB target gene BcL-xL and induction of the pro-apoptotic gene NOXA. In addition to apoptosis, the phosphorylation of T505 negatively regulates many cellular processes such as autophagy, proliferation and cell migration, indicating that this phosphorylation inhibits NF-κB activity and carcinogenesis, which is crucial for the tumour suppressive activity of p65 (Msaki et al., 2011). CHK1 inhibitors are a novel cancer therapy strategy that is currently undergoing clinical trials. Moreover, T505 phosphorylation by CHK1 is necessary for efficient CHK1 activation by ATR, thereby driving dependence on the ATR/CHK1 signalling pathway, which is essential for the survival of cancer cells under high levels of DNA replication stress. Mutations at the p65 T505 phosphorylation site disrupt the DNA replication stress response, resulting in resistance to CHK1 inhibitors (Hunter et al., 2022). Moles et al. (2016) suggest that in p65 T505 mutant mice, sensitivity to liver damage and carbon tetrachloride (CCl4)-induced HCC increases, with significant upregulation of genes associated with proliferation and the cell cycle, as well as significant induction of a gene set related to DNA repair and replication, highlighting the importance of T505 phosphorylation in promoting apoptosis and inhibiting liver cell proliferation.

The S468 site within the TA2 subregion can be phosphorylated by kinases such as GSK-3β, IKKε and IKKβ. Post-translational modification of p65 at S468 mainly regulates the transcriptional activation of NF-κB. In unstimulated Hela cell nuclei, GSK-3β phosphorylates p65 at S468, inhibiting the transcriptional activation of NF-κB (Buss et al., 2004). Conversely, in T cells responding to synergistic stimuli, IKKε can directly phosphorylate p65 at S468 within the nucleus, thereby enhancing downstream gene transcription (Mattioli et al., 2006). Upon stimulation by TNFα and IL1β, IKKβ phosphorylates p65 at S468 in the cytoplasm of various cells, within the IκB-p65 complex, thereby preventing the nuclear entry of NF-κB and downregulating the expression of relevant genes (Schwabe and Sakurai, 2005). Furthermore, 17β-estradiol (E2) promotes the proliferation and tumorigenesis of lung adenocarcinoma cells through oestrogen receptor ERα. E2 increases the expression of TNF-α receptor and TNF-α-triggered NF-κB activity in cells expressing ERα, revealing that GSK-3β enhances TNF-α-induced phosphorylation of nuclear p65 at residues S468 and S536, particularly at S468, facilitating the transcriptional activation of NF-κB. These findings highlight the role of GSK-3β in the crosstalk between E2/TNF-α signalling pathways (Cheng et al., 2021). Zhang et al. (2021) in their study on the anticancer effects of Huanglian Jiedu Decoction (HLJDD) on oral squamous cell carcinoma cell lines, reported that HLJDD specifically inhibits phosphorylation at S468, which could be a reason for its anticancer properties. The diverse activities exhibited upon phosphorylation at the S468 site further underscore the complexity of the post-translational regulation of p65.

T435, located within the TA2 of TAD, is downregulated upon TNFα stimulation, resulting in decreased interaction between p65 and HDAC1 and selective enhancement of NF-κB-dependent gene expression (O’Shea and Perkins, 2010). Yeh et al. (2004) demonstrated that protein phosphatase 4 (PP4) dephosphorylates T435, enhancing cisplatin-induced NF-κB transcriptional activity and consequently reducing cellular resistance and increasing the anticancer effects of cisplatin. Furthermore, the phosphorylation of p65 at T435 inhibits p65 transcriptional activation in response to cisplatin stimulation. Kim et al. (2012) observed an increase in p65-Thr435 phosphorylation in endothelial cells expressing SMI-71 during severe vasogenic oedema triggered by status epilepticus in the piriform cortex. Thus, neutralising TNF-α through soluble TNF p55 receptor (sTNFp55R) infusion was demonstrated to inhibit p65 T435 phosphorylation in endothelial cells, alleviating vasogenic oedema and neuronal damage induced by status epilepticus. Zhou et al. (2013) employed mutagenesis experiments to reveal that the Thr464 residue of p65 is crucial for mitochondrial regulation. Resveratrol activates p65 by phosphorylating Thr464 via the PKC activation of PKG, promoting interaction between p65 and PGC-1a, which, in turn, inactivates PGC-1a, reduces mitochondrial content and induces hepatic steatosis.

### 2.3 Phosphorylation sites at other locations

Phosphorylation sites located at the junction of p65 TAD and RHD are limited, with currently identified sites including S311, S316 and T308. Duran et al. (2003) discovered mice that protein kinase Cζ (PKCζ) can specifically induce p65 S311 phosphorylation in response to TNF stimulation. Phosphorylation at this site plays a crucial role in recruiting p300/CBP to NF-κB and activating downstream IL-6 gene transcription. A study on pneumonia revealed that mice with PKCζ gene knockout exhibited reduced symptoms of lung inflammation following exposure to cigarette smoke (CS) and LPS compared to wild-type mice, as PKCζ facilitates p65 S311 phosphorylation, subsequently activating NF-κB. Hence, PKCζ knockout reduces inflammation (Yao et al., 2010). Wang et al. (2015) demonstrated through mass spectrometry analysis that p65 is phosphorylated at Ser-316 after IL-1β treatment, thereby activating NF-κB transcription and inducing cell proliferation. Fluorescence luciferase assays indicated that CKI inhibitor D4476 significantly decreased NF-κB-induced activity in IL-1β-treated and wtp65-overexpressing 293C6 cells, but had no significant impact on cells overexpressing the S316 mutation. These findings suggest that CKI is the kinase responsible for phosphorylating p65 Ser-316. Additionally, mutation at S316 leads to NF-κB activation and reduced cell growth (Wang et al., 2015). Moreover, P65 T308 can be phosphorylated by phosphatase inhibitor calyculin A treatment. Ma et al. (2017) explored the protein kinase database Scansite for p65 sequence motifs, where PKC is considered the sole presumed kinase target.

## 3 Other post-translational modifications of p65

### 3.1 Acetylation of p65

The post-translational modification of p65 through acetylation has been extensively studied, revealing various inducible acetylation sites. Currently, seven acetylation sites have been identified on p65: K122, K123, K218, K221, K310, K314, and K315. Unlike phosphorylation, which predominately occurs outside the nucleus, acetylation primarily takes place within the nucleus, exerting distinct regulatory functions (Quivy and Van Lint, 2004).

Chen et al. (2001) were the first to demonstrate acetylation modifications on p65. They found that acetylated p65 enhances NF-κB transcriptional activation and its DNA interaction. This enhancement is facilitated by the recruitment of p300/CBP. Acetylation of p65 also disrupts its binding to IκB. However, certain signalling pathways recruit HDAC3 to remove acetylation modifications on p65, facilitating IκB binding. Consequently, this leads to the translocation of p65 to the cytoplasm, thereby terminating NF-κB transcriptional activation. Subsequent studies further confirmed the acetylation of p65 and identified three sites, K218, K221 and K310. Acetylation at K221 enhances p65’s DNA binding, while K310 acetylation enhances its transcriptional activation. Simultaneous acetylation at K221 and K218 counteracts IκB binding (Chen et al., 2002). Additionally, p65’s K122 and K123 can be acetylated by p300/CBP and the p300/CBP-associated factor (PCAF), neutralising their positive charge and thereby reducing NF-κB DNA binding ability. This promotes the recruitment of IκB, leading to the suppression of the pathway’s transcriptional activity. The acetylation at these two sites may synergise with the deacetylation of K218, K221 and K310 (Kiernan et al., 2003). Notably, under TNFα stimulation, endogenous chromatin-bound p65 is acetylated at the K314 site under, demonstrating p300’s acetylation of p65 at K314 and K315, which are two novel acetylation sites (Buerki et al., 2008; Rothgiesser et al., 2010).

### 3.2 Methylation of p65

Protein methylation is another important form of modification, predominately targeting lysine and arginine residues. Lysine methylation can occur in mono-, di- and trimethylated forms, whereas arginine methylation may manifest as symmetrical mono/dimethylation or asymmetrical mono/dimethylation (Murn and Shi, 2017).

Methylation of proteins generally increases their hydrophobicity, thus influencing their activities and interactions with other proteins. Among the enzymes identified to catalyse the methylation of p65 arginine residues, protein arginine methyltransferase 5 (PRMT5) holds prominence, PRMT5 plays a crucial role in normal physiology and cancer development, particularly in haematologic malignancies (Zhu and Rui, 2019). This enzyme catalyses symmetrical dimethylation modifications on five arginine residues of p65, namely, R30, R35, R174, R304, and R330, enhancing downstream gene transcription. Gene chip analysis reveals that mutation at R30A can result in approximately 85% downregulation of PRMT5-induced NF-κB target gene expression (Wei et al., 2013; Harris et al., 2014; Harris et al., 2016). Additionally, SETD6 acts as a lysine methyltransferase, mediating monomethylation of p65 at the K310 site. This modification leads to chromatin binding of p65 in the absence of cellular stimulation, retaining it in the nucleus. Monomethylation at K310 renders p65 inactive, negatively regulating TNF-induced NF-κB activation. Moreover, SETD6 attenuates p65-driven cell proliferation and suppresses inflammatory responses in primary immune cells (Levy et al., 2011). The protein lysine methyltransferase Set7, also known as Set9, selectively monomethylates p65 at K314 and K315, inducing the proteasomal degradation of p65 and thus negatively regulating the NF-κB pathway (Yang et al., 2009). However, methylation at K37 is essential for RelA binding to DNA, facilitating NF-κB target gene transcription by stabilising the binding of NF-κB to its enhancers. Consequently, it facilitates the transcription of downstream NF-κB target genes to regulate the inflammatory response (Ea and Baltimore, 2009).

Under IL-1β stimulation, p65 undergoes reversible methylation at the K218 and K221 sites, significantly affecting its function. Lu et al. (2010) found that monomethylation at K218 and dimethylation at K221 are catalysed by histone methyltransferase NSD1 (nuclear receptor binding SET domain protein 1) in complex with H3K36, thereby activating the NF-κB pathway. Additionally, they demonstrated that FBXL11 mediates the demethylation of K218 and K221, negatively regulating NF-κB transcriptional activity and reducing the proliferation and colony formation of HT29 cancer cells. Guo et al. (2023) conducted animal experiments revealing that histone arginine demethylase JMJD6 attenuates isoproterenol-induced myocardial hypertrophy by interacting with cytoplasmic NF-κB p65 under hypertrophic stimuli, reducing p65 nuclear levels. JMJD6 binds to p65 and demethylates p65 at the R149 residue, inhibiting p65 nuclear translocation, thus inactivating NF-κB signalling and preventing pathological cardiac hypertrophy.

### 3.3 Ubiquitination of p65

Ubiquitin molecules are widely present in eukaryotic cells, and consequently, the ubiquitination of p65 primarily occurs within the nucleus, often triggered by DNA binding. This modification involves a series of reactions mediated by the activation enzyme E1, the binding enzyme E2 and the ligase enzyme E3, with the latter specifically recognising target proteins. Through regulating protein ubiquitination, the E3 participateS in various cellular physiological processes, underscoring its importance in the ubiquitin pathway. Numerous NF-κB ubiquitination-related regulatory factors have been identified, including E3 ligases such as SOCS1 (Maine et al., 2007), PPARγ (Hou et al., 2012), ING4 (Hou et al., 2014), RNF182 (Zeng et al., 2023), ORF73 (Rodrigues et al., 2009), among others.

The suppressor of cytokine signalling 1 (SOCS1) serves as the first identified NF-κB E3 ligase. As a member of the intracellular protein CIS-SOCS (SOCS1-7) family, SOCS1 interacts with the accessory factor copper metabolism MURR1 domain containing 1 (COMMD1) to form a complex with ECS (SOCS1), stabilising the interaction between SOCS1 and p65 and facilitating the ubiquitination of the p65 protein (Maine et al., 2007). SOCS-1 interacts with p65 to regulate the ubiquitination and degradation of p65, a process that is controlled by Pin1, a peptidyl-prolyl cis-trans isomerase. SOCS-1 overexpression downregulates p65 and consequently inhibits NF-κB activity (Anrather et al., 2005). Additionally, ING4, a growth suppressor and member of the chromatin-modifying protein ING family, plays a crucial role in terminating NF-κB activation. Through its plant homeodomain (PHD) motif, ING4 promotes p65 ubiquitination at the K48 site and proteasomal degradation, as well as playing a significant role in the ubiquitination of p65 at the K62 site. However, mutations in the receptor site of p65 at K62 hinder ING4-induced p65 ubiquitination. ING4 not only functions in tumorigenesis but also plays a critical role in the negative regulation of inflammation (Hou et al., 2014). RNF182, identified as a novel tumour-suppressive E3 ubiquitin ligase, induces p65 ubiquitination, suppressing PDL1 transcription and immune suppression in lung adenocarcinoma, thus mitigating cancer progression (Zeng et al., 2023). PPARγ, a peroxisome proliferator-activated receptor, acts as an E3 ligase facilitating p65 ubiquitination and degradation through its RING domain. Deletion of PPARγ in murine embryonic fibroblasts resulted in increased stability of p65. Conversely, overexpression of PPARγ led to ubiquitination of p65 at the K48 site, resulting in a rapid decrease in p65 half-life. Studies on the K28 mutant of p65 revealed a counteractive effect on PPARγ-mediated ubiquitination and degradation. The ubiquitination and degradation of p65 mediated by PPARγ are crucial as they terminate the pro-inflammatory response and xenograft tumour progression orchestrated by p65 (Hou et al., 2012). The E3 ligase protein FBXW2, which contains F-box and WD repeat domains, plays a key role in ubiquitinating p65 at the K122 site, leading to decreased expression of SOX2, a pivotal transcription factor in stem cell maintenance. FBXW2 mediates p65 degradation both *in vitro* and *in vivo*, resulting in decreased cell proliferation and migration, ultimately impacting stemness, breast tumor growth, and paclitaxel resistance (Ren et al., 2022).

In addition to the classical ubiquitin-proteasome degradation pathway, p65 degradation can also occur through the ubiquitin-lysosome pathway. TRAF7 facilitates p65 K29 ubiquitination, leading to its degradation within lysosomes. Furthermore, TRAF7 promotes cell death and inhibits p65 activity (Zotti et al., 2011). Fan et al. (2009) identified K195 as a potential ubiquitination receptor site in the p65 protein, essential for polyubiquitination mediated by p65 overexpression. This residue downregulates TNFα-induced NF-κB activation, affecting downstream gene expression. Li et al. (2012) utilised mass spectrometry analysis and identified acetylated lysine residues K122, K123, K314 and K315 as ubiquitin receptor sites, responsible for p65 polyubiquitination. Additionally, other lysine residues, including K65, without acetylation have been reported, necessitating further investigation into their specific functions.

### 3.4 Glycosylation of p65

O-GlcNAcylation represents a reversible, transient and dynamic protein post-translational modification, under the regulation of O-GlcNAc transferase (OGT) and O-GlcNAcase (OGA). Yang et al. (2008) identified T322 and T352 as O-GlcNAcylation sites on p65 through mutagenesis experiments and mass spectrometry analysis. O-GlcNAcylation at these sites inhibits the interaction between NF-κB and IκB, facilitating increased nuclear translocation of p65 and subsequent NF-κB transcriptional activation. This mechanism may contribute to sustained NF-κB activation under hyperglycaemic conditions. Further studies have demonstrated that O-GlcNAcylation of T322 and T352 in colonic tissue modulates p65 promoter recruitment, thereby enhancing the activation of NF-κB signalling and promoting the development of colitis and related cancers (Yang et al., 2015). Furthermore, p65 can undergo O-GlcNAcylation at T305, S319, S337, Th352 and S374. Among these sites, O-GlcNAc modification at p65 T305 contributes to NF-κB transcriptional activity without inhibiting its interaction with IκBα, representing a crucial site required for O-GlcNAcylation (Allison et al., 2012; Ma et al., 2017).

Recent research has unveiled novel p65 O-GlcNAcylation serine sites at S550 and S551, which drive NF-κB activation and the pancreatic ductal adenocarcinoma (PDAC) phenotype. Mutations affecting these sites impact PDAC cell proliferation, anchorage-independent growth and migration. The interplay between OGT and p65 suggests potential combined clinical significance in PDAC progression, hinting at OGT inhibition as a novel approach for future PDAC therapeutic strategies (Motolani et al., 2023). Turmeric has shown promise in ameliorating diseases associated with high O-GlcNAcylation. Studies indicate that turmeric inhibits overall O-GlcNAcylation in liver cells, along with reducing the O-GlcNAcylation of proteins such as p65 and ChREBP-c. This leads to NF-κB signalling pathway deactivation, ultimately reducing oxidative stress and inflammation levels in hepatic cells (Lee et al., 2019). Furthermore, p65 O-GlcNAcylation enhances the pulmonary metastasis of cervical cancer cells by upregulating the expression of C-X-C chemokine receptor 4 (CXCR4) (Ali et al., 2017).

### 3.5 Poly-ADP-ribosylation of p65

Poly (ADP-ribosyl)ation represents a rapid and transient post-translational protein modification catalysed by poly ADP-ribose polymerase 1 (PARP1). This modification, observed in eukaryotic cells, is reversible and commonly implicated in inflammatory disease models. Liu et al. (2012) demonstrated that in LPS-stimulated mouse macrophages Ana-1 or Raw264.7, the interaction between PARP1 and p65, coupled with p65 poly (ADP-ribosyl)ation, promotes NF-κB transcriptional activation, leading to increased expression of IL-1β and IL-18 genes. The ERK pathway regulates PARP-1 activation, with inhibitors of this pathway blocking the interaction between PARP1 and ERK1/2, the phosphorylation of PARP1 and p65 poly (ADP-ribosyl)ation. This suggests that PARP1 relies on ERK to regulate its activity and activate NF-κB, indicating that the ERK-PARP1-p65 pathway in macrophages enhances the inflammatory process of infectious diseases. Another study by Zerfaoui et al. (2010) revealed that the ADP-ribosylation of p65 is crucial for its interaction with nuclear export protein Crm1 (exportin 1). Effective nuclear retention of p65 NF-κB under TLR4 stimulation requires PARP-1 enzyme activity, thereby modulating the expression of downstream cytokine genes in smooth muscle cells.

### 3.6 S-nitrosylation of p65

Nitration reactions primarily target tyrosine residues of proteins. Liquid chromatography coupled with nanoelectrospray mass spectrometry analysis revealed specific nitration of p65’s Y66 and Y152. Upon cellular exposure to nitric oxide stimulation, rapid nitration reactions occur at these sites, leading to the nuclear export of p65 and inactivation of NF-κB (Park et al., 2005). In respiratory epithelial cells and macrophages stimulated with cytokines, p65 becomes a target of NO. Through mutagenesis experiments, a conserved Cys-38 within the RHD DNA binding site was identified as the S-nitrosylation site, inhibiting NF-κB-dependent gene transcription. Additionally, the nuclear levels of S-nitrosylated p65 inversely correlated with the DNA binding of the p50-p65 heterodimer. Furthermore, the S-nitrosylation of p65 is also suggested to be dependent on NOS2 activity (Kelleher et al., 2007). Nitration reactions have implications in various diseases, particularly in the nervous system. N-methyl-D-aspartate (NMDA) receptor-mediated excitotoxicity leads to cell death, which negatively impacts brain function. Additionally, NMDA activation induces NF-κB activation and nuclear translocation, promoting the transcriptional activation of pro-inflammatory genes such as IL-1β. Endothelial nitric oxide synthase (eNOS) participates in p65 S-nitrosylation and subsequent NF-κB inhibition in the cerebral cortex, exerting neuroprotective effects (Caviedes et al., 2021).

### 3.7 Glutathionylation of p65

The S-glutathionylation of p65 involves the reversible formation of mixed disulfide bonds between its cysteine residues and glutathione (GSH). *In vitro* mass spectrometry analysis revealed S-glutathionylation modifications at cysteine residues 38, 105, 120, 160, and 216 of p65 following oxidation and nitrosation stress. Among these residues, Cys38, 160 and 216 have been identified as S-glutathionylation sites in NF-κB p65. The GSH/ROS-dependent glutathionylation of p65 has the potential to mediate NF-κB inactivation. CD spectroscopy and tryptophan fluorescence measurements indicate that S-glutathionylation does not alter p65’s secondary structure but affects its tertiary structure (Lin et al., 2012; Qanungo et al., 2014). Moreover, GRx-dependent S-glutathionylation of p65-NF-κB likely leads to NF-κB inactivation and enhanced hypoxic cell apoptosis (Qanungo et al., 2007). Clinical data analysis reveals a correlation between high NF-κB expression and shorter overall survival in patients with non-small cell lung cancer (NSCLC), indicating its tumour-promoting function. S-glutathionylation negatively regulates NF-κB by interfering with its DNA binding activity. Furthermore, under the influence of oxidants, the S-glutathionylation of NF-κB enhances pulmonary inflammation. Therefore, S-glutathionylation serves as a crucial factor in the regulation of NF-κB, wherein elevated levels of NF-κB are linked to shorter. Overall, the expression of NF-κB is considered a significant adverse prognostic marker in NSCLC (Zhang et al., 2005).

### 3.8 S-sulfhydration of p65

S-sulfhydration, is a post-translational modification initiated by the formation of hydrogen sulfide residue (-SSH) within protein cysteine residues. The S-sulfhydrylation site of p65 is C38, and modification at this site inhibits p65’s DNA binding, suppressing downstream gene transcription and nuclear localisation (Perkins, 2012; Tamura et al., 2012). The S-sulfhydrylation of NF-κB appears to be a physiological determinant of its anti-apoptotic transcriptional activity. Sen et al. (2012) observed that the TNF-α stimulation of cystathionine gamma-lyase (CSE) transcription results in the generation of hydrogen sulfide (H_2_S), which facilitates the binding of p65 at C38, thereby enhancing its interaction with the co-activator RPS3. This interaction subsequently augments its association with several anti-apoptotic gene promoters. CSE-deficient mice, unable to S-sulfhydrate p65, exhibit reduced NF-κB target gene activity, TNF-α hypersensitivity and increased cell death. Consequently, the S-sulfhydration of NF-κB appears to be an essential modification for p65’s transcriptional impact on anti-apoptotic genes. CSE is an H_2_S-generating enzyme that promotes NF-κB nuclear translocation through S-sulfhydrating at C38 of the NF-κB p65 subunit mediated by H_2_S, resulting in increased IL-1β expression and H_2_S-induced cell invasion, suggesting that S-sulfhydrylation of p65 may directly contribute to cancer metastasis (Wang Y. H. et al., 2019). Increased H_2_S levels induce the S-sulfhydrylation of wild-type p65 plasmid-transfected THP-1-derived macrophages in a concentration-dependent effect. Mutation of p65 C38 abolishes H_2_S-induced S-sulfhydrylation, which inhibits NF-κB activation by targeting the free thiol group on C38 of the NF-κB p65 subunit and consequently suppresses macrophage inflammation (Du et al., 2014).

### 3.9 S-sulfenylation of p65

Protein S-sulfenylation refers to the oxidation of cysteine thiol group (Cys-SH) to cysteine sulfinic acid (Cys-SOH), which is a reversible process. Utilising the DAZ-2 labelling method, it was observed that sulfite (SO_2_) donor combined with oleic acid (OA) treatment significantly increased the sulfenylation of NF-κB p65 in A549 cells compared to cells treated with OA alone or untreated. Mutation experiments on C38 revealed that C38S mutation prevented SO_2_-induced S-sulfenylation, indicating that SO_2_ enhances p65 S-sulfenylation at C38. S-sulfenylation of p65 at C38 suppresses NF-κB p65 nuclear translocation, DNA binding activity and ICAM-1 promoter recruitment in A549 cells, thereby inhibiting NF-κB p65 phosphorylation and activation. This mechanism elucidates how endogenous SO_2_ mitigates acute lung injury induced by OA by modulating pulmonary inflammation (Chen et al., 2017).

### 3.10 Prolyl isomerisation of p65

Proline 65 undergoes isomerisation catalysed by Pin1, with the catalytic site located at position 255. Upon binding to the Thr254-Pro motif phosphorylated by p65, Pin1 catalyses p65 isomerisation, inhibiting its interaction with IκBα and promoting p65 nuclear localization and protein stability. This enhances NF-κB nuclear translocation and DNA binding activity. Inhibition of Pin1 in breast cancer tissue suppresses NF-κB activation (Ryo et al., 2003), suggesting Pin1 as a potential target for controlling aberrant NF-κB activation in cancer and related diseases.

### 3.11 ISGylation of p65

ISGylation is a reversible covalent modification process similar to the ubiquitin-like modifier system SG15 and the ubiquitin-proteasome system, involving a series of enzyme cascades divided into two stages. In this process, ISG15 binds to target proteins through a dual-glycine sequence and undergoes covalent modification known as ISGylation. Studies demonstrate that ISGylation of NF-κB p65 in quiescent endothelial cells (EC) reduces endothelial cell inflammation, which is reversible. However, TNFα and EC endotoxin stimulation decrease p65 ISGylation, promoting its serine phosphorylation by reducing its binding to the phosphatase WIP1. Mechanistically, the SCF (Skp1-Cul1-F-box) E3 ubiquitin ligase complex SCFFFBXL19 has been identified as a novel ISG15 E3 ligase that targets and catalyses p65 ISGylation. Depletion of F-box and leucine-rich repeat protein 19 (FBXL19) increases p65 phosphorylation and EC inflammation, suggesting a negative correlation between p65 ISGylation and phosphorylation. Furthermore, transgenic mice overexpressing EC-specific FBXL19 exhibited reduced lung inflammation and severity of experimental acute lung injury (Li et al., 2023).

### 3.12 SUMOylation of p65

A small ubiquitin-related modifier (SUMO) mediates the SUMOylation modification of p65. Unlike ubiquitination, SUMOylation does not target proteins for proteolysis but rather regulates protein function in various cellular processes. Elevated levels of SUMO1-related p65 SUMOylation in cancer tissues may enhance the nuclear translocation of p65 and activation of the NF-κB signalling pathway, promoting HCC invasion and metastasis, thus accelerating HCC progression (Liu et al., 2020).

### 3.13 Alkylation of p65

Sesquiterpene lactones (SLs), found in various medicinal plants of the Asteraceae family, exhibit anti-inflammatory properties. García-Piñeres et al. (2001) conducted mutational experiments and discovered that SLs directly inhibit NF-κB binding to DNA by alkylating the p65 subunit at Cys38, thereby suppressing NF-κB activation at the final step of transduction. Furthermore, they demonstrated the selectivity of SLs towards p65 compared to N-ethylmaleimide (García-Piñeres et al., 2004). These findings highlight the potential of natural products like SLs to develop into novel anti-inflammatory agents.

## 4 Interaction of post-translational modifications

Post-translational modifications interact in a complex manner, with one modification potentially enhancing or inhibiting another. The protein p65 effectively regulates the transcriptional activity of NF-κB through various post-translational modifications and their interactions.

### 4.1 Phosphorylation and acetylation

The intricate relationship between phosphorylation and acetylation of p65 is evident. Phosphorylation at different sites on p65 has been demonstrated to enhance acetylation at K310. For instance, phosphorylation of p65 at the S276 site promotes its interaction with p300/CBP, leading to the acetylation of K310 on p65 and thus enhancing its transcriptional activity (Nihira et al., 2010; Brasier et al., 2011). Conversely, mutations at the S276 gene in a mouse model revealed that dephosphorylated p65 readily binds to HDAC3, suppressing the transcription of numerous downstream NF-κB genes and inhibiting the expression of non-NF-κB-regulated genes through epigenetic mechanisms (Dong et al., 2008). Phosphorylation at the S536 site is necessary for the acetylation of p65 at the K310 site (Rajendrasozhan et al., 2010). In response to RSV replication, the acetylation of p65 at K310 is induced, with this acetylation being contingent upon phosphorylation at S276, thereby modulating the transcriptional elongation of inflammatory cytokines during RSV infection (Brasier et al., 2011). Additionally, phosphorylation of p65 at T435 weakens its interaction with HDAC1, resulting in reduced HDAC1 recruitment and increased p65 acetylation (Zhang et al., 2021). Moreover, the phosphorylation of p65 at the S311 site inhibits HDAC5-mediated deacetylation of p65 K310, promoting NF-κB activation (Zhou et al., 2022). These studies collectively demonstrate the significant role of p65 phosphorylation in its acetylation process.

### 4.2 Phosphorylation and ubiquitination

The interaction between ubiquitination and phosphorylation modifications is well established. Phosphorylation at residue S276 shields p65 from SOCS-1-mediated ubiquitination and subsequent proteolytic degradation (Nihira et al., 2010). Conversely, inhibiting phosphorylation of p65 at sites S205, S276 and S281 directly facilitates p65 ubiquitination (Hochrainer et al., 2012). Moreover, under certain inducible conditions, p65 phosphorylation can induce ubiquitination. For example, in the presence of CK1γ1, the interaction between p65 and CUL2 is significantly enhanced. Phosphorylation of p65 at residue S536 by CK1γ1 facilitates p65 ubiquitination and degradation through the E3 ubiquitin ligase CUL2 and COMMD1, thereby inhibiting NF-κB transcriptional activity and downstream signalling pathways, ultimately negatively regulating the RIG-1 signalling-mediated innate immunity (Wang et al., 2014). Additionally, phosphorylation of p65 at residue S468 induced by TNFα promotes the association of p65 with components of the polyubiquitin ligase complex that mediates p65 ubiquitination, such as COMMD1 and cullin2, thereby enhancing ubiquitination expression (Geng et al., 2009). Subsequent research has demonstrated that under stress conditions, the ubiquitination of p65 in association with COMMD1 is not reliant on phosphorylation at S468 following administration of aspirin. Further elucidation through additional investigation is necessary to fully understand the interplay between these two modifications (Thoms et al., 2010).

### 4.3 Effects among other post-translational modifications

Different chemical modifications can interact with each other to impact the overall functionality and activity, showcasing a range of diversity and complexity. Phosphorylation, along with acetylation and ubiquitination, may have inhibitory effects on methylation. The monomethylation of p65 at K310 can be recognised by the anchor protein repeat sequence of histone methyltransferase GLP. Under basal conditions, this recognition reduces the open chromatin state of p65 target genes through GLP-mediated histone H3K9 dimethylation. However, PKCζ phosphorylates p65 at S311, disrupting the binding of GLP to p65 K310 monomethylation. Consequently, this phosphorylation weakens the suppression of target genes (Levy et al., 2011).

Li et al. (2012) employed mass spectrometry analysis to investigate the ubiquitination of p65 at K122, K123, K314, and K315. They found that these sites are also subject to acetylation. This simultaneous dual modification of acetylation and ubiquitination at the same site suggests a potential competitive inhibitory effect, thus contributing to gene-specific regulation of nuclear NF-κB function. Experimental studies manipulating the activity of p300 through modulation, knockdown, or the use of the P300 inhibitor (C646) have shown that FBXW2 ubiquitinates the p65 site K122 based on p300-induced acetylation. The acetylation of p65 by p300 can hinder the ubiquitination of p65 induced by FBXW2 (Ren et al., 2022).

The involvement of the E3 ubiquitin ligase TRAF6 in innate immune signaling pathways is modulated by arginine methylation facilitated by PRMT1. PRMT1 directly interacts with TRAF6, resulting in its methylation. Methylation of arginine residues on TRAF6 diminishes its ubiquitin ligase function, consequently suppressing NF-κB activation (Tikhanovich et al., 2015). Acetylation at residue 65 has the potential to hinder methylation events. Yang et al. (2010) identified that enhancing the acetylation of K310 inhibits the methylation of K314 and K315 regulated by Set9, while simultaneously reducing the ubiquitination regulated by methylation. This mechanism enhances the stability of the interaction between p65 and chromatin and consequently augments the transcriptional activity of NF-κB. Interestingly, the regulation of methylation at these two sites does not affect the acetylation of K310.

Glycosylation enhances acetylation, and inhibits phosphorylation. Upon TNF stimulation, OGT localises on chromatin, and O-GlcNAc modification becomes essential for p300-mediated p65 K310 acetylation. The O-GlcNAcylation of p65 at T305 and S319 sites enhances the CBP/p300-dependent activation acetylation at the K310 site of p65, thereby facilitating NF-κB transcriptional activation (Allison et al., 2012; Ma et al., 2017). Advanced glycation end products (AGEs) are sugar-modified biomolecules, activating the AGE-RAGE signalling pathway that triggers the phosphorylation of p65 at three specific residues: T254, S311 and S536. These modifications are crucial for collagen I gene transcription, suggesting a potential link to ageing. Additionally, the glycosylation and phosphorylation of p65 are interrelated (Peng et al., 2016). Treatment with N-acetylglucosamine or O-(2-acetamido-2-deoxy-D-pyranose glucosamine) amino-N-phenylaminomethanesulfonate prevents the TNF-α-induced increase in NF-κB p65 O-GlcNAc modification, thereby inhibiting p65 phosphorylation at the S536 site and the subsequent transcription and expression of inflammatory mediators. Conversely, OGT overexpression increases O-GlcNAcylation, resulting in elevated expression of p300, IKKα and IKKβ, thereby promoting IKK-mediated p65 phosphorylation at the S536 site and facilitating NF-κB activation (Xing et al., 2011). Moreover, p65 phosphorylation at T308 may impair O-GlcNAcylation at T305 (Ma et al., 2017). Furthermore, mutations such as S550A and S551A in p65 may compromise the intensity and duration of S536 phosphorylation in PDAC (Motolani et al., 2023).

The phosphorylation of the S276 site on p65 and the SUMO1-mediated p65 SUMOylation at the S276 site are interdependent processes that reinforce each other. In patients with HCC, the phosphorylation level of p65 is elevated in cancerous tissues, concomitant with elevated SUMO1 expression, while SUMO2/3 remains unaffected. Additional clinical evidence highlights a positive association between SUMO1 and phosphorylated p65, suggesting that SUMO1-mediated p65 SUMOylation and p65 phosphorylation at S276 jointly enhance the vitality and invasiveness of liver cancer cells, consequently reducing apoptosis (Jiang et al., 2024).

Additionally, there is a possibility of substitution between modifications, where S-nitrosylation may replace S-sulfhydration. Sen et al. (2012) unveiled that the nitrosylation of p65 C38 displaces S-sulfhydration, culminating in the inhibition of the NF-κB response. Upon TNF-α treatment, P65 undergoes initial S-sulfhydration modification by H_2_S, activating corresponding promoters and exerting an anti-apoptotic effect. Subsequently, NO generated by iNOS induces S-nitrosylation of p65, counteracting the activating effect induced by S-sulfhydration modification.

## 5 Conclusion and perspective

This study comprehensively elucidates 14 distinct modes of post-translational modifications of p65, encompassing phosphorylation, acetylation, methylation, ubiquitination and glycosylation, alongside multiple kinases catalysing these post-translational modifications. Rather than acting in isolation, these modifications interact in a synergistic or antagonistic manner, forming a complex regulatory network to exert their effects. Early research has suggested that differences in the phosphorylation levels between different sites on p65 are crucial for the NF-κB-specific regulation of downstream target gene expression and proposed a “phosphorylation code” for p65 (Vermeulen et al., 2003). Subsequent investigation by Lanucara et al. (2016) unveiled the dynamic phosphorylation of endogenous p65 at multiple sites post TNF-α stimulation, providing insights into the temporal dynamics of endogenous p65 phosphorylation in SK-N-AS neuroblastoma cells in response to TNF-α exposure. Additionally, this study provides evidence for identifying different p65 “codes” and demonstrates that p65-mediated transcription does not solely involve one modification corresponding to one effect, but rather encompasses a combination of different modifications.

Aberrant activation of the NF-κB signalling pathway is a hallmark of various cancer cell lines and solid tumours, underscoring the significance of downregulating NF-κB activity as a therapeutic strategy. Post-translational modifications play a pivotal role in NF-κB regulation, and selectively inhibiting protein kinases that promote NF-κB activity hold promise as a supplementary approach in cancer therapy. For instance, inhibitors of PKA such as H-89 (Arun et al., 2009) and curcumin analogues like UBS109 (Zhu et al., 2014) exhibit promising inhibitory effects on head and neck SCC growth, while the Pim-1 inhibitor SMI-4a (Wang J. et al., 2019) mitigates LPS-induced acute lung injury, and CHK1 inhibitors (Hunter et al., 2022) emerge as novel candidates in cancer therapy clinical trials. It is noteworthy that a single kinase can target multiple sites on p65, with contrasting effects resulting from these modifications. For example, GSK-3β phosphorylates T254, S536 and S468 on p65, with phosphorylation of T254 and S536 promoting NF-κB transcriptional activation while phosphorylation of S468 inhibits it. This underscores the intricate interplay within p65 post-translational modification networks and emphasises the need for a nuanced understanding when using inhibitors. In conclusion, a comprehensive exploration of p65 post-translational modifications not only enhances our understanding of NF-κB regulatory mechanisms in inflammation, immune responses and cancer but also paves the way for the identification of novel therapeutic targets for various diseases. This facilitates the development of drugs with enhanced specificity and minimal side effects targeting the NF-κB pathway.
